# Food Habits of the Wolf in a Low-Density Territory in the Northeast of Trás-os-Montes (Portugal)

**DOI:** 10.3390/ani15060873

**Published:** 2025-03-19

**Authors:** Samuel Lemos, Luis Llaneza, Armando Pereira, Aurora Monzón

**Affiliations:** 1 Centro Nacional de Reprodução do Lince-Ibérico (CNRLI), 8375-082 Silves, Portugal; 2 A.RE.NA Asesores en Recursos Naturales S.L., c/Perpetuo Socorro, 12 Entlo 2B., 27003 Lugo, Spain; llaneza@arenatural.com; 3 GIBE (Evolutionary Biology Research Group), Departamento de Bioloxia, Área Zoología, Campus da Zapateira s/n, 15071 A Coruña, Spain; 4 Department of Forestry Science and Landscape Architecture (CIFAP), School of Agrarian and Veterinary Sciences (ECAV), University of Trás-os-Montes e Alto Douro (UTAD), P.O. Box 1013, 5000-801 Vila Real, Portugal; argo1269@hotmail.com (A.P.); amonzon@utad.pt (A.M.); 5 Centre for the Research and Technology of Agroenvironmental and Biological Sciences (CITAB), Inov4Agro, University of Trás-os-Montes e Alto Douro (UTAD), P.O. Box 1013, 5000-801 Vila Real, Portugal

**Keywords:** *Canis lupus signatus*, trichology, scats, diet, conservation

## Abstract

The study of carnivores’ diets is an important conservation tool, which can minimize conflicts with different stakeholders and provide proper substantiations for management measures. The main aim of this study was to understand the diet of a small, isolated pack named Mogadouro Sul, present in the Northeast of Trás-os-Montes (Portugal). Scat analysis was the method used to determine what wolves consumed. The specific origin of all collected fecal samples (n = 78) was confirmed by molecular analysis. The results, expressed in frequency of occurrence (FO) showed that domestic animals were the most frequent food category in the wolf’s diet (78.3% FO), with a special incidence in goats (40.6% FO), although wild ungulates (roe deer and wild boar) also accounted for 21.7% FO of the diet. The study pack presented a diet diversity (H′) of 0.65 and a food niche breadth (B′) of 0.55. Food availability did not appear to be a limiting factor, and the wolf’s presence in the study area could be explained by changes in land use and increased infrastructure. This trophic behavior may threaten the conservation of this pack due to the persecution it may face.

## Simple Summary

This study was conducted to investigate the occurrence of historic wolf packs registered in the influence area of Sabor reservoir and determine the trophic habits of the pack detected (Mogadouro Sul pack). We discuss how the Iberian wolf population in this area could be affected by the availability of food resources and the influence it may have on the shepherd-wolf conflict. We analyzed the diet composition examining the scats collected in the study area between 2015 and 2017, which were genetically confirmed. This pack mainly consumes domestic animals rather than wild ungulates although they are present throughout the study area. We attribute these findings to the fact that it is a small pack with limited predatory capacity. These feeding habits may affect public opinion and human tolerance towards wolves. Therefore, even in territories with a scarce presence of wolves, the measures to prevent attacks on livestock cannot be neglected, to its safeguard.

## 1. Introduction

Studies indicate that 61% of the world’s largest carnivores are threatened with extinction [1]. Therefore, resolving conflicts between Mankind and predators is an important matter to avoid or mitigate deadly persecution, which is the most common social response [2]. This can lead to a massive reduction in carnivore’s geographic ranges or to their extinction [3]. Nevertheless, in recent decades there has been a recovery of the large carnivores in Europe, partly due to the exodus of the rural population and agricultural land abandonment [4] associated with a rewilding landscape occurring naturally [5,6] and aided by legal protection. The Iberian wolf is an example of this, as it is internationally protected by the Berne Convention, the Habitats Directive and CITES [7], and its subspecies, *Canis lupus signatus*, is classified as “Endangered” in the Portuguese Red Data Book [8], and it has been legally protected since 1988 (Law n° 90/88 of 13 August and Decree-Law 54/2016 of 2 August). However, the concentration of wolf packs in some European regions, where wolf populations have been able to recover and grow, has led the European Commission to re-evaluate their conservation status [9], with an approved proposal to downgrade it from ‘strictly protected’ to ‘protected’ under the Bern Convention.

The global tendency towards urbanization creates a social duality concerning the acceptance of great carnivores such as the wolf. Urban society is manifestly more favorable toward wolves than the rural society [4,10,11,12,13], which lives in proximity to wildlife and bears the costs of crop and livestock losses. In Portugal, livestock predation by the Iberian wolf is the primary cause of human–carnivore conflict, despite the species having lost more than 80% of its historical range during the 20th century [14] and currently inhabiting the highland regions of the north and center of the country [8].

Several studies on the wolf’s diet have been conducted in Portugal based on the analysis of scats but generally without resorting to genetic confirmation due to the high associated costs. Most results show a strong dependence on domestic animals, with the main prey changing according to local densities [15,16,17]. Livestock grazing systems vary according to livestock species and breed. Cows are raised near rural areas, primarily in intensive systems. Semi-extensive and extensive systems are used more with sheep and goats. The last system is characterized by multi-owner herds dominated by autochthonous breeds, grazing extensively at the tops of mountains, far from shelter, and rarely confined within fences or barns, which may make animals particularly vulnerable to predation [18].

The compensation system for wolf predation consists of damage payment (Decree-Law 54/2016 of 25 August). The Institute for Nature Conservation and Forestry (ICNF) pays compensation for documented losses [19], covering the total market value. However, payment is limited to a specified number of attacks by civil year, and it is conditioned, for example, to the number of guardian dogs, according to the size of herds (Decree n° 335/2017, 6 November), and/or their confinement to barns at night.

**Figure 1:**
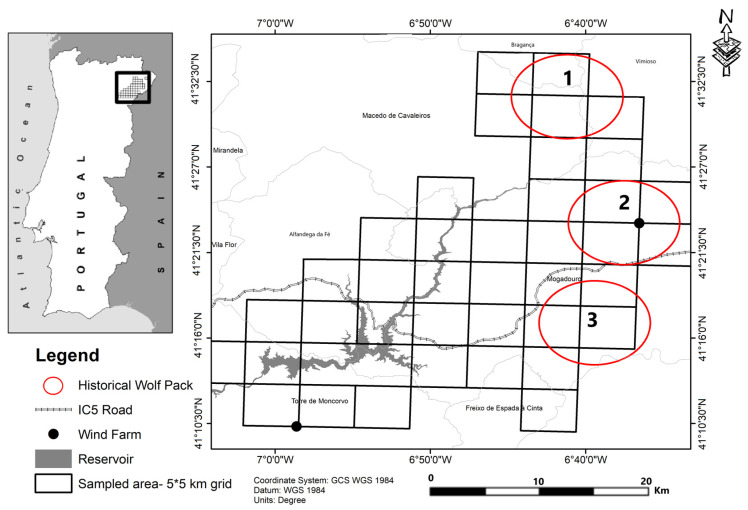
Location of the study area; sampled area (5 × 5 km grid); main infrastructure, and the placement of the packs historically present: (1) Talhinhas; (2) Mogadouro Norte; and (3) Mogadouro Sul.

According to Torres and Fonseca (2016) [20], the Portuguese wolf population is distributed by four nuclei: Peneda/Gerês, Alvão/Padrela, Bragança, and Douro’s South. Since the last census to the present time [17], some areas of these nuclei have experienced a fast transformation of the landscape derived from rural abandonment and fragmentation. The latter is motivated mainly by the construction of new communication routes, wind farms, and the installation of dams, with consequences for the structure and dynamics of the wolf packs. This applies to the Bragança nucleus where since 2003, a new road was built (IC5), two wind farms were installed (with a total of six wind turbines) and the Baixo Sabor Dam hydroelectric project [21] was built (2013), with approximately 3000 ha of flood area along 69.6 km. Fire is also a common factor of disturbance, and it should be noted that four major fires have occurred in the area since 2013 [22]. These events influenced mainly the center-south zone of the nucleus, where this study took place (Figure 1), with three historical wolf packs.

This study was conducted in the influence area of Sabor reservoir and intended to investigate (i) the occurrence of the historic wolf pack registered in the study area, (ii) if the natural prey, wild boar and roe deer, which occur in the region and currently expanding in Portugal are being incorporated in the wolf diet [23,24]. We evaluate whether food resources availability (domestic and wild ungulates) could influence the viability of wolf packs, and how the wolf diet could trigger shepherd–wolf conflicts.

## 2. Materials and Methods

### 2.1. Study Area

This study was developed at the area of influence of the Baixo Sabor reservoir (Figure 1), located at the Sabor River, northeast Portugal (41°40′–41°10′ N, 7°10′–6°25′ W), between 109 and 997 m above sea level (a.s.l.), with the highest altitudes on a set of ridges on the south (southeast side). The climate is Mediterranean with a continental tendency, being characterized by warm, dry summers and concentrated precipitation in the autumn and spring seasons. Precipitation ranges between 480 mm/yr and 1000 mm/yr, and average annual temperatures vary between 15 °C and 10 °C [25].

According to the Biogeographical Map of Portugal by Costa et al. (1998) [26] this area belongs to the Carpetan–Leonese sector, Lusitanian–Duriensean subsector, where the vegetation consists of perennial sclerophyllous woodlands dominated by oak (*Quercus* spp.) with the presence of junipers (*Juniperus oxycedrus*) [27]. Nowadays, these woodlands are largely substituted by arable land, chestnut groves, and Mediterranean cultivated plants. Pine plantations (mainly *Pinus pinaster*) and small areas with eucalyptus (*Eucalyptus* spp.) are common in this landscape in mosaic, where the typical Mediterranean vegetation is mostly relegated to the slopes. In percentage terms of land use and occupation, it is dominated by agriculture (43%), forests (30%, open forest and shrublands), pastures (26%), and other areas (1%). Livestock farming is mainly based on small ruminants (sheep and goats) and, to a lesser extent, on cattle, where traditional grazing systems in the extensive regime remain. The wild boar (*Sus scrofa*) represents the main wild species followed by the roe deer (*Capreolus capreolus*), with both being widespread.

### 2.2. Data Collection

This study’s method for determining the Iberian wolf’s diet was scat analysis because it is a non-invasive technique that is very effective for collecting information on carnivores’ diet [16] and allows homogeneous sampling, with an accuracy of 80–90% [28,29]. The wolf scats were collected from three samplings per year (spring, summer, and autumn) between 2015 and 2016. Additionally, a final sampling was made in the winter of 2017 in the most relevant transects. The study area was divided into 39 squares (25 km^2^ each) using the Universal Transverse Mercator (UTM) grid system of 10 × 10 km, which resulted in a total of 975 km^2^. A total of 104.23 km was traveled on rural and forest roads (minimum of 2 km and maximum of 3.75 km) by vehicle, at a speed under 20 km/h. At intersections, smaller roads branching off from the main roads were surveyed on foot for up to 50 m in each direction [30].

During the data collection, we considered the scat’s morphology, size, and shape, among other characteristics attributed to wolf scats [31]. All possible wolf scats were photographed, collected, placed in plastic bags, and labeled. The scats were collected using personal protective equipment: gloves and face masks. The coordinates of the location of each scat were registered (using GPS). Scats with low certainty of species identification, or which were found deteriorated, were not collected. In the laboratory, scats were placed in sterile plastic bottles and filled with 96% ethanol.

Due to the difficulties of macroscopic distinction between wolf and dog scats, non-invasive genetic methods were used [31]. Therefore, later scats were preserved in ethanol (96%) and sent to the Research Centre in Biodiversity and Genetic Resources (CIBIO), an independent research unit that performed the genetic molecular analysis for the species identification, which followed the DNA extraction method proposed by Gerloff et al. (1995) [32] and the indications of Godinho et al. (2015) [33].

A total of 94 samples were collected, of which 78 (Table 1) were confirmed by genetic identification (89%) as wolf samples. Of these scats, 9 did not have any material or food remains that allowed an accurate identification of the consumed species and were discarded, resulting in 69 valid samples for dietary analysis.

According to Pimenta et al. (2005) [7] the historical wolf population in this study area consists of three confirmed packs and one likely to exist. However, despite efforts to visit the area of all these packs, all genetically identified wolf scats were only collected in the area occupied by the Mogadouro Sul pack. This monitoring, between 2015 and 2017, was performed within the Integrated Environmental Monitoring Program (PIMA) project. Through molecular genetic analysis for individual identification, four individuals were recognized, three males and one female, in 2015 (with reproduction); two individuals in 2016, the reproductive pair of 2015 (without reproduction) [34]; and in 2017 the same pair and a new female [35].

### 2.3. Scats Analysis

For each scat, a superficial observation of the sample and its contents was performed. Then, scats were dissected and based on the general characteristics the possible ingested species were registered. Bone fragments and nails were kept separately to, if possible, be analyzed later and compared with reference material. Vegetation, fruits, invertebrates, or soil were not considered food items because their intake appears to be involuntary. The adequate hairs were selected, and the slides were prepared according to the specialized bibliography [36,37,38,39,40,41,42,43,44,45,46,47].

The procedure for preparing the slides and the cuticular pattern was as follows: clean the hair properly with 96% commercial ethyl alcohol and dry it on absorbent paper; apply a layer of hair lacquer on the slide and place the hair for 10 min, which is enough time to imprint the cuticular pattern on the slide, before removing the hair with the help of tweezers.

The cuticular pattern of the hair provides most of the characteristics necessary to identify the species to which they belong, and it is not affected in the digestive tract of predators [43]. Therefore, in this study, only the cuticular pattern was observed. The prepared slides were observed on an OLYMPUS BX50F optical microscope (Olympus Optical Co., Ltd., Tokyo, Japan) and compared with our reference collection which included the hairs of species considered as potentially consumed prey.

### 2.4. Wolf Diet Analysis

To obtain a direct measure of the undigested remains in the scats, the diet composition was quantified in terms of frequency of occurrence (FO) because it is one of the most used and biologically expressive indexes referring to wolf diet quantification [48]. To categorize the importance of each prey, we used the classification proposed by Ruprecht (1979) [49] based on the frequency of occurrence: basic resource (FO ≥ 20%); regular resource (5% < FO ≤ 20%); supplementary resource (1% < FO ≤ 5%); occasional resource (FO ≤ 1%).

Since this method may overestimate the frequency of small prey, we converted the FO values to biomass consumption (BC) using the linear regression method of Floyd et al. (1978) [50] revised and readjusted by Weaver (1993) [51]:

y=0.439+0.008x

where *y* represents the biomass in kilograms of the food type consumed per scat collected, and *x* represents the average live weight of each prey species identified [51]. By multiplying each *y* by the total number of scats per prey species, we estimated the total biomass of each prey type [16]. The average live body masses used to calculate the biomass consumed were as follows: domestic goat: 40 kg [52]; domestic sheep: 60 kg [53,54]; domestic cow: 200 kg [55]; domestic dog: 20 kg [56]; roe deer: 24 kg [56]; and wild boar: 70 kg [16].

Dietary Diversity (*H*′) was calculated using the Shannon–Wiener Index:

$$H^{\prime }=-\sum [pi\;\log\left(pi\right)]$$

where *pi* corresponds to the proportion of prey species *i* in the diet. When all preys are equally abundant, dietary diversity reaches the maximum value and is equal to *Log N*, where *N* is the number of preys identified.

The standardized food niche breadth (*B*′) was estimated using the standardized Levins’ index:

$$B^{\prime }=\frac{\left(1/\sum {pi}^{2}\right)-1}{N-1}$$

where *N* is the number of prey identified and *pi* is the proportion of each prey species in the diet, as mentioned above. The index ranges from 0 to 1, 0 when there is only one prey being consumed, and 1 when all prey is consumed equally, which reflects the degree of specialization of the diet.

We also performed a seasonal analysis of the diet in which data were divided into two smaller groups corresponding to two periods: (1) reproductive season (winter and spring) and (2) non-reproductive season (summer and fall). The food categories found were divided into two groups: (1) domestic animals and (2) wild ungulates. Seasonal comparisons of the diet were performed using a reference table in which the Chi-square (χ^2^) test was applied, first to test whether significant differences occurred between the total diet composition and the two periods and then to detect individually whether each prey group differed between the two periods analyzed. These comparisons were performed using IBM SPSS Statistics 24 statistical software.

### 2.5. Domestic Small Ruminants’ Trend

To understand the condition of agricultural activity of small ruminants breeding (goats and sheep), we analyzed the data available from two different official sources, DGAV (Direção de Serviços de Alimentação e Veterinária) (Table A1 in Appendix A) and INE (Instituto Nacional de Estatística) (Table A2 in Appendix A), regarding the variation in number of animals and farms, in the parish where the wolf was present and in the surrounding ones, in the period of 2012–2019.

The data from DGAV is presented by year in the period of 2012–2019; however, this does not present the data of goat and sheep separately but as a group, as small ruminants. The INE data, despite being available separately for goats and sheep, only referred to the years 2009 and 2019. For this reason, they had to be analyzed and interpreted separately.

## 3. Results

It is important to highlight that only mammalian items were found in the scats and the scat analysis only reveals what wolves consumed, which does not necessarily correspond to what they hunted, since cases of scavenging of carcasses are quite frequent [48]. The Mogadouro Sul pack based its trophic habits on six species. Four of these species are domestic animals: goats, sheep, cattle, and dogs. The remaining two correspond to the only wild ungulates present in the study area: roe deer and wild boar.

### 3.1. Global Diet Composition

Domestic animals were the most dominant food category of the diet of the Mogadouro do Sul pack (Table 2), reaching a frequency of occurrence of 78.3% and a biomass consumption of 77.1%; more specifically the domestic ungulates with 68.1% FO and 70% BC. Goats were the most important species in their diet (40.6% FO and 36.7% BC), followed by sheep (24.6% FO and 26.4% BC) and, therefore, were considered basic food resources. The wild ungulates did not have much importance in the pack diet (21.7% FO and 22.9% BC); however, the wild boar and the roe deer were considered as regular food resources (15.9% FO and 5.8% FO, respectively; 18.6% BC and 4.3% BC, respectively). Dog was also considered a regular food resource (10.1% FO and 7.1% BC). Cattle was the least frequent species (2.9% FO) and was considered a supplementary food resource; however, when expressed in biomass consumption (6.9% BC) this species showed a higher percentage than roe deer which has the lower biomass consumption (4.3% BC) input value of all species consumed.

Considering the annual cycle, the diet diversity was 0.65 and the food niche breadth was 0.55.

### 3.2. Seasonal Diet Composition

Regarding seasonal variation (Table 2), summer showed the highest frequency of domestic animals, comprising nearly 90% of the diet. On the other hand, winter was the season in which domestic animals were less frequent (<55%).

Goats were the only species that constituted a basic resource in all seasons, being more frequent in autumn. Sheep were also an important part of this pack’s diet but were only a basic food resource in summer when they had a higher % FO. Dog was considered a regular food resource throughout the year (except in winter) mainly in the non-reproductive season. Regarding scats with cow remains, these were only found in the non-reproductive season, being considered a supplementary food resource in summer and a regular one in autumn. Concerning wild ungulates, the wild boar was the only species present in all seasons, being consumed regularly, except in spring when it was most consumed (basic resource). Roe deer was more consumed in winter, which was considered a basic food resource; however, in summer it was only a supplementary resource.

The comparison between the reproductive and non-reproductive periods, using the Chi-Square test, demonstrated significant differences (*p* < 0.05) regarding total prey consumption. Regarding domestic prey, there were significant differences between the two seasons (*p* < 0.05), and their consumption was much higher in the non-reproductive one. On the other hand, no significant differences (*p* > 0.05) were found in wild prey consumption.

Both diet diversity and food niche breadth had a small variation during the different seasons (Table 2). The diet of the Mogadouro Sul pack was more diverse in autumn (H′ = 0.62) and less diverse in the spring (H′ = 0.55), reflecting that the data differences were not relevant. This pack showed a greater specialization in goat consumption in the spring since the lowest value of food niche breadth was reached in this season (B′ = 0.45). In winter the prey consumption was not as specialized as in the other seasons because in winter the food niche breadth reached the highest value (B′ = 0.53). The differences between B′ data were also not relevant between seasons.

### 3.3. Domestic Small Ruminants’ Trend

**Figure 2:**
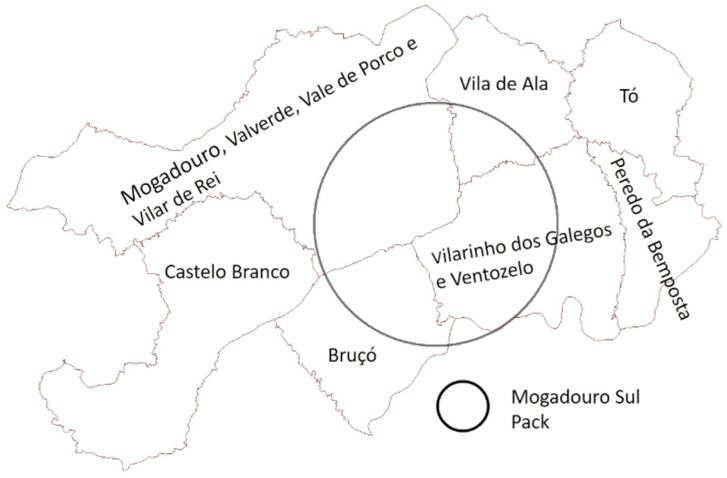
Parishes surrounding the Mogadouro Sul pack area.

As the domestic small ruminants were the most dominant food category of the diet of the Mogadouro do Sul pack, we tried to understand the evolution of the livestock activity, between 2012 and 2019, in the surrounding parishes to the pack area (Figure 2).

**Figure 3:**
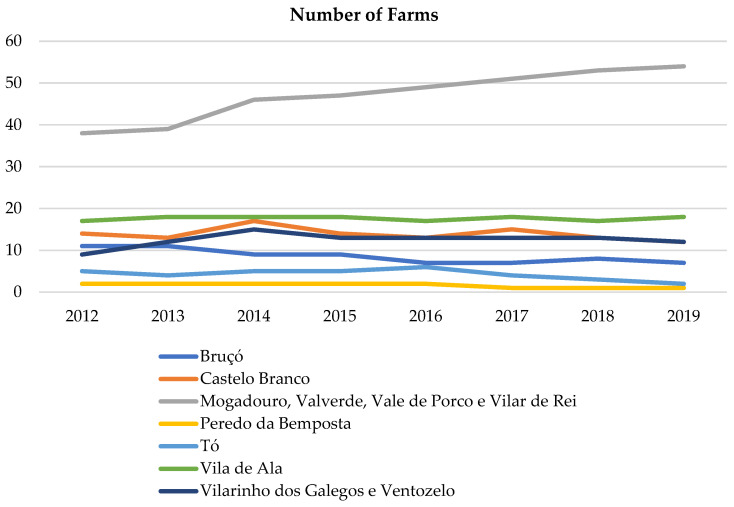
Number of farms between 2012 and 2019 in the pairs where the wolf pack was present and in the surrounding ones (data from DGAV).

In Figure 3 (DGAV data from 2012 to 2019) is possible to observe that the number of farms tends to maintain over time, except in the case of the parish of Mogadouro (Group of parishes of Mogadouro, Valverde, Vale do Porco e Vilar de Rei), which increased, and in addition, it has the largest number of farms.

**Figure 4:**
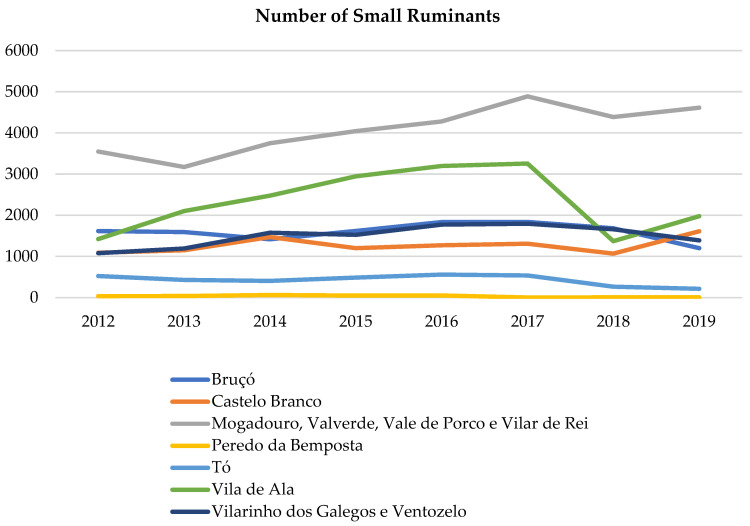
Number of small ruminants between 2012 and 2019 in the pairs where the wolf pack was present and in the surrounding ones (data from DGAV).

Regarding the number of small ruminants (Figure 4), we observed that, between 2012 and 2017, some parishes maintained a similar number of animals, while others even experienced an increased the number of animals with an equivalent number of farms. Nevertheless, since 2017 there has been a decrease in the number of animals, followed by a slight increase. Vilar de Ala was the parish with the most significant loss of small ruminants.

As already mentioned, with DGAV data it is not possible to differentiate how the observed trend affects small ruminants separately.

Data from INE (Table 3 and Table 4) allowed us to understand the evolution of small domestic ruminants in two groups (goat and sheep); however, they only allowed comparing data from two specific years, 2009 and 2019. The data indicate that there was no significant variation in the number of farms, but in all parishes except those of Mogadouro, there was a large decrease in the number of sheep from 2009 to 2019. The opposite happened with the number of goats, in which most villages had a significant increase comparing 2009 with 2019.

Considering the data from both sources, it can be observed that the number of farms has remained similar over time, while the number of small ruminants decreased (DGAV). This had a major impact on the number of sheep; however, there was an increase in the number of goats (INE). Therefore, these preferential trophic resources for wolves did not decline over time.

## 4. Discussion

Our study shows that domestic animals were the most frequent prey (78.3% FO and 77.1% BC) of the Mogadouro do Sul pack, particularly goat, in this pack trophic behavior, which agrees with the results found in many of the studies carried out in the Iberian Peninsula [16,48,56,57,58,59,60,61,62]. This may be related to the traditional grazing system in extensive regimes, which is common in this studied region. This type of livestock farming system makes the herds more accessible to wolves. The fact wolves consume more goats than sheep may be related to sheep herding systems observed in this area. Sheep tend to form tighter herds and graze in areas closer to villages, where they are monitored by shepherds and guard dogs [16,62]. On the other hand, cattle are not very accessible to wolves because, in this region, the number of cows in free ranging is insignificant, and cattle are often kept closer to villages in confined areas [16] resulting in a small consumption. In addition to domestic ungulates, it also verified consumption of domestic carnivores, in this case dogs, such as in other studies carried out in the Iberian Peninsula [48,58,59,63,64,65], which refers to it as a supplementary or regular trophic resource, but in our study a regular one (10.1% FO).

Wild prey consumption was found to be less significant in this pack’s feeding habits (21.7% FO and 22.9% BC) as has been observed in other studies [16,48,56,62]. Since these species are the wolf’s natural prey, a high consumption would be expected based on data from some regions of the Iberian Peninsula [56,57,63,64,65,66,67,68], in most of Europe [69,70,71,72,73,74,75,76,77] and North America [78,79,80]. In this region, the only existent wild ungulate species are wild boar and roe deer. In similarity with other studies, wild boar was the most frequent wild prey followed by roe deer [68]. The results referring to % FO and % BC were quite similar in all species present in the wolf’s diet, except in one case, in which the consumption of cattle was higher than roe deer with the % BC values, which did not happen with % FO.

According to our results, this pack consumed only six categories of prey, and two of them were found in much higher proportions (goats and sheep), the reason diet diversity was low (H′ = 0.65). One consequence of this pack food habit is a relatively low value of food niche breadth (B′ = 0.55), which reflects the degree of diet specialization in a singular food category, the domestic ungulates, particularly in goats, as was also found in other studies [16,62].

To keep a proper trophic strategy, predators must select the most profitable prey. In other words, the prey that maximizes the energy gains by time spent, risk of injury and probability of successful capture on the acquisition of each prey [74,81]. Over time, it was verified that the existence of seasonal variation patterns in the consumption of domestic ungulates, depending on their availability, which is correlated to their abundance and to the livestock farming system. Mogadouro Sul pack consumed significantly more domestic animals in summer/autumn than in winter/spring, which was also found in other regions of Europe [56,57,82]. In summer/autumn, there is a greater availability and accessibility of domestic ungulates, due to different herd practices, to births of offspring, and due to herds’ free grazing habits, especially small ruminants, which increase their susceptibility to wolf attacks [57,75,77,83]. During the non-breeding season, the wolf explored all domestic species, including cattle, which were not part of its diet in the breeding season. This season variation may be due to the reduced availability of domestic species in winter, as herding becomes more restricted to areas closer to the villages [48,57].

In contrast to other Iberian wolf packs, in the Mogadouro Sul pack, there were no statistical differences in wild ungulates consumption during the different seasons. The wild boar was consumed regularly and constantly, although in the spring there was a slightly higher consumption, agreeing with other studies [65,74], due to the presence of young individuals [62]. Otherwise, the other wild prey, roe deer, was only consumed in winter and summer. Despite the non-statistical differences, it was possible to observe an increase in the consumption of wild ungulates in those seasons that favor the capture of animals, which may reinforce the opportunistic behavior of this predator [56,57,62,63,65,68,73,74].

Considering the diet over the year, it was less diverse in spring (H′ = 0.55), which has also been reported in other studies [48,56], while autumn presented the highest value (H′ = 0.62), which means that it was the time of year in which a greater number of prey classes were consumed [62]. The bigger vulnerability of domestic ungulates in spring/summer leads to less proportional consumption of different food categories, which translates into a lower B′ value (B′ = 0.45). In winter, the highest B′ value (B′ = 0.53) can be explained by a more proportional consumption of food categories. However, our study did not reveal strong seasonal differences, presenting very close values throughout seasons referring to H′ and B′. Despite the expansion of wild ungulates [23,24], there was evidence of preference for domestic ungulates over the years.

Since the studied pack was composed of a small number of individuals (maximum of four individuals in 2015) it is expected that the predatory success of this pack would be limited, thereby they will be foraging prey that requires spending less energy, specifically domestic animals like goats. According to Lanszki et al. (2012) [73], Wagner et al. (2012) [74], and Imbert et al. (2016) [76], wolves in packs consume more wild prey than dispersing individuals, which in turn consume more frequently domestic species. This occurs because, as expected, the dispersing wolves have a much lower hunting success compared to the packs, so they choose prey with less effective defensive behavior.

Despite the total sample size of the present study appearing to be reduced, when compared to similar studies, considering that it is a single pack with a small number of individuals, the significant amount of genetically confirmed scats makes it representative in determining its food habits.

The results of this study are conditioned by a decrease in the number the historical packs present in the area and by the current existence of only one pack with a reduced number of individuals. In addition, it is important to highlight that we can only stipulate that this pack may be representative of other small packs found in areas with similar habitats and with similar availability of livestock. Since the Portuguese population is considered marginal, it is reasonable to assume that the average number of animals per pack is lower than that estimated in more central areas of the species’ distribution (mean value of six wolves/pack, in breeding season; and mean value of three adult/subadult wolves per pack being the minimum value) [84]. In the Bragança nucleus, the total mean number of wolves was estimated at 4 (±1.3 sd), with the average number of offspring equal to 1.9 (±1 sd) according to Pimenta et al., 2023 [84].

One of the factors identified as a potential threat to wolf conservation is the lack of food resources, both wild and domestic prey. In the case of domestic prey, this is linked to rural exodus, which has led to a decline in livestock densities [16,20]. In our study area, considering the two sources of data analysis on small ruminants’ herds (DGAV and INE), it was clear that the number of animals remained relatively stable during the analyzed period (2012–2019). While the number of sheep has declined, the number of goats has increased, resulting in an overall stability in small ruminant populations over time (Figure 4). Since goats are the prey most consumed by this pack, the wolf’s main domestic trophic resource in the study area does not appear to be at risk. Nevertheless, this type of trophic habit is a cause of conflict with shepherds, resulting in part from the husbandry practice. In the study area, many shepherds no longer experience the pressure of wolf attacks as in the past years, leading to the belief in their non-existence. Consequently, there is a disregard for herd protection practices [85], among which regarding the number of guard dogs required per unit of livestock, due to the cost–benefit ratio. Even in areas with low wolf density, a single attack can trigger widespread conflict, making poaching one of the main factors affecting the presence of wolf in this area, as has also happened in others wolf nucleus [18,86]. Since the existing compensation system is currently the primary tool for mitigating livestock losses caused by wolf attacks [20], it is necessary to reinforce it, as it helps reduce hostility from rural communities toward this predator. However, precautions should be taken in its management to avoid creating dependency [87].

The decrease in the number of historical packs in this area, comparing our monitoring with the previously published census [7], could be explained, in addition to interaction with humans, by changes in land use and increased infrastructure [84]. These changes reduce the availability of suitable areas for wolf refuge and reproduction, increase fragmentation and reduce habitat quality, conditioning the distribution of this species in humanized landscapes [88]. Facing all these threats, the Action Plan for the Conservation of the Iberian Wolf in Portugal (Dispatch n.° 9727/2017) points to the need to maintain a habitat in a favorable state of conservation, avoiding any conflict with shepherds through the environmental education of rural populations and encouraging coexistence with this carnivore. Improving livestock system techniques and the use of effective protection measures [16,69,74,76,86,89] are essential for reducing herd losses given the feeding habits of this pack and others with similar conditions, according to Pimenta et al., 2023 [84].

On the basis of our results and considering the negative population trend in one considerable area of the Bragança nucleus, which can result in significant changes in habitat from the implementation of numerous infrastructures, we consider it important to include landscape attributes in future investigations [88,90,91].

## 5. Conclusions

This study contributed to the reinforcement of scientific knowledge about the wolf diet in Portugal, and how it may be a tool for conservation, highlighting the importance of improving prevention measures, regardless of the constant presence or not of the wolf in a territory, preventing or reduce conflicts between this predator and local communities.

The main conclusion of this study is that the Mogadouro Sul pack had a high consumption of domestic ungulates, particularly goats. This reflected a similar trophic behavior to the other packs studied in Portugal. However, it diverges with the generality of the wolf diet studies (in which there was more consumption of wild prey) in Europe or North America.

Although the consumption of domestic prey was predominant, some seasonal differences were found in the diversity of prey consumed and frequency of occurrence. This may happen due to the small size of the pack (only four adult individuals in 2015 and two in 2016) which uses strategies that optimize feed acquisition, taking advantage of the traditional herd grazing systems, more common on goats and sheep.

Food availability did not appear to be a limiting factor for wolves in our study area, with only one pack of the three historically present. It will be important to investigate, in the future, which other human and environmental factors are at the origin of the situation observed in the occurrence of the species, to incorporate compensation and adaptation measures into a dynamic landscape.

It is crucial, in the integrated management and conservation measures plan, to seriously focus on measures that enable the coexistence between wolf populations, their prey, humans, and their subsistence activities. Additionally, it is important to extend the knowledge of the wolf’s dependence on scavenging carcasses whether from the deposition of dead animals due to livestock practices or from dead wild ungulates by poaching.

## Figures and Tables

**Table 1:** Number of scats collected per identified wolf.

Wolf Identification	Number of Scats		
	2015	2016	2017
Male 1 (cub)	3	-	-
Male 2	6	6	8
Male 3 (cub)	1	-	-
Female 1	8	15	7
Female 2	-	-	1
Without individual identification	5	13	5
Total number of scats (78)	23	34	21

**Table 2:** Determination of percentage of frequency of occurrence (FO), percentage of biomass consumption (BC), diet diversity (H′) and food niche breadth (B′) of the Mogadouro do Sul pack feeding towards different types of prey, in each season of the year and in the annual cycle.

	Winter			Spring			Summer			Autumn			Annual Cycle		
Prey	n	FO	BC	n	FO	BC	n	FO	BC	n	FO	BC	n	FO	BC
Domestic Animals	6	54.5	55.9	7	63.6	58.1	28	90.3	90.0	13	81.3	79.2	54	78.3	77.1
Dog	0	0.0	0.0	1	9.1	6.3	4	12.9	9.1	2	12.5	8.3	7	10.1	7.1
Domestic Ungulates	6	54.5	55.9	6	54.5	51.8	24	77.4	80.9	11	68.8	70.9	47	68.1	70.0
Goat	4	36.4	35.1	4	36.4	32.5	13	41.9	38.3	7	43.8	37.6	28	40.6	36.7
Sheep	2	18.2	20.8	2	18.2	19.3	10	32.3	34.9	3	18.8	19.1	17	24.6	26.4
Cow	0	0.0	0.0	0	0.0	0.0	1	3.2	7.7	1	6.3	14.1	2	2.9	6.9
WILD UNGULATES	5	45.5	44.1	4	36.4	41.9	3	9.7	10.0	3	18.8	20.8	15	21.7	22.9
Wild boar	2	18.2	22.6	4	36.4	41.9	2	6.5	7.6	3	18.8	20.8	11	15.9	18.6
Roe deer	3	27.3	21.4	0	0.0	0.0	1	3.2	2.4	0	0.0	0.0	4	5.8	4.3
Total	11	100	100	11	100	100	31	100	100	16	100	100	69	100	100
H′	0.58			0.55			0.60			0.62			0.65		
B′	0.53			0.45			0.46			0.51			0.55		

**Table 3:** Number of farms and sheep in 2009 and 2019 (data from INE), in the parish where the wolf pack was present and in the surrounding parishes.

Parishes	Farms 2009	Farms 2019	Sheep 2009	Sheep 2019
Bruçó	1	2	1744	1447
Castelo Branco	8	3	1558	923
Peredo da Bemposta	0	1	174	73
Tó	0	0	528	227
Group of parishes of, Valverde, Vale de Porco e Vilar de Rei	11	18	3399	3798
Group of parishes of Vilarinho dos Galegos e Ventozelo	1	6	1428	1183
Vila de Ala	5	3	1584	827

**Table 4:** Number of farms and goats in 2009 and 2019 (data from INE), in the parish where the wolf pack was present and in the surrounding parishes.

Parishes	Farms 2009	Farms 2019	Goats 2009	Goats 2019
Bruçó	12	8	18	129
Castelo Branco	15	11	180	115
Peredo da Bemposta	3	4	0	3
Tó	4	2	0	0
Group of parishes of Mogadouro, Valverde, Vale de Porco e Vilar de Rei	37	47	767	1023
Group of parishes of Vilarinho dos Galegos e Ventozelo	11	14	190	614
Vila de Ala	19	12	213	399

## Data Availability

The data presented in this study are available on request from the corresponding author.

## Appendix A

**Table A1:** Number of farms and small ruminants between 2012 and 2019 in the pairs where the wolf pack was present and in the surrounding parishes (data from DGAV).

Year	Parishes	Farms	Animals
2012	Bruçó	11	1615
	Castelo Branco	14	1089
	Mogadouro, Valverde, Vale de Porco e Vilar de Rei	38	3547
	Peredo da Bemposta	2	34
	Tó	5	521
	Vila de Ala	17	1420
	Vilarinho dos Galegos e Ventozelo	9	1079
2013	Bruçó	11	1590
	Castelo Branco	13	1148
	Mogadouro, Valverde, Vale de Porco e Vilar de Rei	39	3172
	Peredo da Bemposta	2	35
	Tó	4	427
	Vila de Ala	18	2099
	Vilarinho dos Galegos e Ventozelo	12	1191
2014	Bruçó	9	1415
	Castelo Branco	17	1468
	Mogadouro, Valverde, Vale de Porco e Vilar de Rei	46	3750
	Peredo da Bemposta	2	61
	Tó	5	402
	Vila de Ala	18	2475
	Vilarinho dos Galegos e Ventozelo	15	1574
2015	Bruçó	9	1616
	Castelo Branco	14	1199
	Mogadouro, Valverde, Vale de Porco e Vilar de Rei	47	4042
	Peredo da Bemposta	2	48
	Tó	5	483
	Vila de Ala	18	2945
	Vilarinho dos Galegos e Ventozelo	13	1524
2016	Bruçó	7	1834
	Castelo Branco	13	1269
	Mogadouro, Valverde, Vale de Porco e Vilar de Rei	49	4278
	Peredo da Bemposta	2	50
	Tó	6	559
	Vila de Ala	17	3195
	Vilarinho dos Galegos e Ventozelo	13	1771
2017	Bruçó	7	1837
	Castelo Branco	15	1307
	Mogadouro, Valverde, Vale de Porco e Vilar de Rei	51	4888
	Peredo da Bemposta	1	3
	Tó	4	536
	Vila de Ala	18	3255
	Vilarinho dos Galegos e Ventozelo	13	1793
2018	Bruçó	8	1683
	Castelo Branco	13	1067
	Mogadouro, Valverde, Vale de Porco e Vilar de Rei	53	4386
	Peredo da Bemposta	1	5
	Tó	3	264
	Vila de Ala	17	1370
	Vilarinho dos Galegos e Ventozelo	13	1658
2019	Bruçó	7	1201
	Castelo Branco	12	1611
	Mogadouro, Valverde, Vale de Porco e Vilar de Rei	54	4612
	Peredo da Bemposta	1	5
	Tó	2	212
	Vila de Ala	18	1975
	Vilarinho dos Galegos e Ventozelo	12	1387

**Table A2:** Number of farms, sheep and goats in 2009 and 2019 (data from INE), in the parish where the wolf pack was present and in the surrounding ones.

Year	Parishes	Sheep Farms	Sheep	Goat Farms	Goats
2009	Bruçó	1	1744	12	18
	Castelo Branco	8	1558	15	180
	Peredo da Bemposta	0	174	3	0
	Tó	0	528	4	0
	Group of parishes of Mogadouro, Valverde, Vale de Porco e Vilar de Rei	11	3399	37	767
	Group of parishes of Vilarinho dos Galegos e Ventozelo	1	1428	11	190
	Vila de Ala	5	1584	19	213
2019	Bruçó	2	1447	8	129
	Castelo Branco	3	923	11	115
	Peredo da Bemposta	1	73	4	3
	Tó	0	227	2	0
	Group of parishes of Mogadouro, Valverde, Vale de Porco e Vilar de Rei	18	3798	47	1023
	Group of parishes of Vilarinho dos Galegos e Ventozelo	6	1183	14	614
	Vila de Ala	3	827	12	399
